# Systematic review and meta-analysis of the prevalence and determinants of exclusive breastfeeding in the first six months of life in Ghana

**DOI:** 10.1186/s12889-023-15758-w

**Published:** 2023-05-19

**Authors:** Shamsudeen Mohammed, Ibrahim Yakubu, Abdul-Ganiyu Fuseini, Abdul-Malik Abdulai, Yakubu H. Yakubu

**Affiliations:** 1 MedicineDepartment of Non-communicable Disease Epidemiology, Faculty of Epidemiology and Population Health, London School of Hygiene and Tropical Medicine, London, UK; 2 Department of Paediatrics and Child Health, University of Otago, Wellington, New Zealand; 3 Department of Nursing, Nursing and Midwifery Training College, Gushegu, Ghana; 4 School of Nursing and Midwifery, Deakin University, Melbourne, Vic Australia; 5 Department of Nursing, Nurses’ and Midwives’ Training College, Tamale, Ghana; 6 School of Clinical Sciences, Department of Nursing, Auckland University of Technology, Auckland, New Zealand; 7 Department of Intensive Care Unit, Tamale Teaching Hospital, Tamale, Ghana

## Abstract

**Background:**

Exclusive breastfeeding is a public health priority in sub-Saharan Africa. However, systematic reviews on its determinants in Ghana remain scarce. Therefore, we systematically reviewed the prevalence and determinants of exclusive breastfeeding in children 0–6 months in Ghana.

**Methods:**

We conducted systematic searches in Embase, Medline, and Africa-Wide Information from the databases’ inception until February 2021 for studies that assessed the prevalence and determinants of exclusive breastfeeding in children 0–6 months in Ghana. Random-effects meta-analysis was used to estimate the pooled prevalence of exclusive breastfeeding and narrative synthesis to summarise the determinants. We calculated the proportion of total variability that was due to between study heterogeneity using I² statistics, and Egger’s test assessed publication bias. The review is registered with PROSPERO, CRD42021278019.

**Results:**

Out of the 258 articles identified, 24 met the inclusion criteria. Most of the included studies were cross-sectional and were published between 2005 and 2021. The pooled prevalence of exclusive breastfeeding (EBF) among children 0–6 months in Ghana was 50% (95%CI 41.0–60.0%). The prevalence was higher in rural areas (54%) than in urban areas (44%). Several factors were identified as facilitators of EBF, including older maternal age, self-employment, unemployment, living in a large house, being a house owner, giving birth in a health facility, non-caesarean delivery, adequate antenatal attendance, counselling services, participation in support groups, adequate knowledge about EBF, positive attitude towards EBF, and higher maternal education among rural dwellers. Additionally, having an average birthweight facilitated EBF. Barriers to EBF were also identified, including higher maternal education among urban dwellers, less than three months of maternity leave, maternal HIV-positive status, the experience of partner violence, lack of access to radio, inadequate breastmilk production, lack of family support, having a partner who wants more children, counselling on complementary feeding, healthcare worker recommendation of complementary feed, single marital status, and infant admission to neonatal intensive care units.

**Conclusion:**

In Ghana, EBF rates are low, with only about half of all children aged 0–6 months breastfed exclusively. A multi-dimensional approach is required to tackle the diverse sociodemographic, obstetric, and infant-related issues that hinder EBF practice in Ghana.

**Supplementary Information:**

The online version contains supplementary material available at 10.1186/s12889-023-15758-w.

## Background

Optimal breastfeeding of infants and young children is a public health priority in sub-Saharan Africa because of the numerous benefits it affords newborns, both in short and long term [1, 2]. It is well documented that exclusive breastfeeding boosts a newborn’s immune system, guards against gastrointestinal and lower and upper respiratory tract infections, including pneumonia and asthma, and decreases the frequency of otitis media episodes and hospitalisation [1, 3–5]. Breastfed children also have a lower risk of obesity, diabetes mellitus, hypertension, and childhood leukaemia [2, 6]. As a result, the World Health Organization (WHO) recommend the initiation of breastfeeding within one hour after birth and the practice of exclusive breastfeeding for the first six months of a newborn’s life. Mothers are encouraged to introduce safe and sufficient complementary foods after the first six months, while breastfeeding is continued for up to two years and beyond [7].

Despite the benefits and recommendations for exclusive breastfeeding, the global prevalence is suboptimal [8]. Even in sub-Saharan Africa, where nearly all mothers breastfeed, only a small proportion of mothers practise exclusive breastfeeding for the first six months after giving birth. For example, a geospatial analysis in 2019 revealed that only 18 of the 46 countries in sub-Saharan Africa are on track to achieving the World Health Assembly 2030 target of 70% prevalence of exclusive breastfeeding in the six months after birth [9]. In Ghana, the prevalence of exclusive breastfeeding has declined drastically from 63% in 2008 to 43% in 2018 [10]. The decline has been attributed to several factors, including traditional practices such as the introduction of water and other homemade preparations to newborns and the indiscriminate advertisement of breastmilk substitutes [11]. In addition, many studies in Ghana have reported on several maternal and child factors and family sociodemographic characteristics that influence the practice of exclusive breastfeeding, including antenatal and postnatal attendance, infant birthweight, place of delivery, mode of delivery, maternal education, occupation, misconceptions about exclusive breastfeeding, and pressure from mothers-in-law, traditional birth attendants or grandmothers [10, 12–18].

A preliminary search of the literature revealed that no systematic reviews or meta-analyses have been conducted to summarise the prevalence and determinants of exclusive breastfeeding among infants younger than six months in Ghana. A review will highlight current trends in the prevalence of exclusive breastfeeding and the factors that promote or hinder the practice to inform healthcare policies and design pragmatic interventions to tackle the declining exclusive breastfeeding rate. Annually, malnutrition costs the Ghanaian economy about $2.6 billion or 6.4% of the gross domestic product [19]. Such expenditure is significant given Ghana’s low-income level and resource-constrained economy. Policies to scale up breastfeeding can mitigate this cost and reduce the incidence of malnutrition, especially in the first six months of life, since exclusive breastfeeding provides children with complete and adequate nutrition needed for optimal growth and development [20]. This study, therefore, aimed to conduct a systematic review and meta-analysis of the prevalence and factors associated with exclusive breastfeeding in the first six months of an infant’s life in Ghana.

## Methods

The protocol for this systematic review was registered with PROSPERO (Number CRD42021278019) [21] and reported following the Preferred Reporting Items for Systematic Reviews and Meta-Analyses (PRISMA) guidelines [22].

### Search strategy

We searched Embase, Medline, and Africa-Wide Information using a combination of subject headings and free text terms from the databases’ inception until February 2021. The database searches were carried out using the following terms: exclusive breastfeeding; exclusive breast feeding; exclusively breastfed; breastfed exclusively; breastmilk only; breastmilk alone. The breastfeeding terms were combined with terms for Ghana and the sixteen regions of the country. The search was initially created in the Medline database (see supplementary Table 1 for the search plan) before being translated for the other databases using subject headings suitable for each database’s thesaurus. Boolean and proximity operators were used to combine synonyms and keywords describing the main concepts, which were then searched as free-text terms. To include various variations of the terms, truncations and wildcards were used. In addition, we searched for additional studies in the references of studies that met the inclusion criteria and articles that cited them.

### Eligibility criteria

The outcome investigated was exclusive breastfeeding, defined as feeding an infant with only breastmilk and no other liquids or solids except for oral rehydration salt, drops, and syrups (vitamins, mineral supplements, or medicines) in the first six months after birth [7]. We included quantitative observational and experimental studies that assessed the prevalence, predictors, determinants, and factors associated with exclusive breastfeeding in the first six months of an infant’s life in Ghana. However, studies that pooled data from several countries, including Ghana but did not present Ghana-specific estimates were excluded. Studies that investigated the effects of exclusive breastfeeding on mortality, morbidity, nutrition status, and health-related outcomes were excluded, except where the prevalence of exclusive breastfeeding in Ghana was estimated. In addition, we excluded studies that assessed the factors associated with exclusive breastfeeding in children older than six months and those that were not conducted in Ghana. Dissertations, systematic reviews, meta-analyses, and conference papers with no adequate information on the study’s methods were excluded. Because we aimed to conduct a meta-analysis of the prevalence of exclusive breastfeeding, we included qualitative studies that reported the prevalence of exclusive breastfeeding in Ghana. Studies were not excluded based on the language or date of publication. A summary of the inclusion and exclusion criteria is presented in Supplementary Table 2.

### Selection and data extraction

The Mendeley citation manager was used to combine articles from the databases before they were exported and deduplicated using Rayyan QCRI systematic review management software [23]. The titles and abstracts of the retrieved studies were screened by authors SM and IY for relevance, and SM then obtained the full texts of any studies that passed the initial screening. SM and IY read the full text of the articles to establish their eligibility using the same criteria. Studies that met the predefined inclusion criteria were retained for critical appraisal and data extraction. Authors SM and IY extracted information on the author’s name (s), study location, study design, sample size, sampling technique, description of study participants, data source and measurement of exclusive breastfeeding, and factors associated with exclusive breastfeeding. Disagreements during the screening and data extraction were resolved through discussions.

### Critical appraisal of studies

The methodological quality of the included studies was evaluated using the Joanna Briggs Institute’s (JBI) critical appraisal tools. The JBI appraisal tools are intended to comprehensively evaluate the methodological quality of included studies and ascertain the extent to which each study has addressed the potential for bias in its design, conduct, and analysis. This information is then used to synthesise and interpret the findings of the studies [27]. A variety of JBI appraisal tools are available for the quality assessment of different studies. We used appraisal tools for analytical cross-sectional studies [24], cohort studies [25], and randomised controlled trials (RCTs) [26]. The cross-sectional appraisal tool has eight items, while the tools for cohort studies and randomised controlled trials consist of 11 and 13 items, respectively. In this study, the original tools were applied without modification. “Yes” or “No” were used to indicate whether a criterion was present or absent, respectively, while “unclear” was used when authors did not provide sufficient details to allow for criterion evaluation. The appraisal tool has no distinct cut-off points. It is recommended that authors use cut-off values appropriate for their study to determine whether a study is of low, moderate, or high quality. However, cut-off scores are generally discouraged because items of the critical appraisal tool are not equally weighted [28]. As a result, we did not exclude articles based on the results of the quality appraisal. Nonetheless, cross-sectional studies with scores of eight or seven, six to four, and three or less were considered to have a low, moderate, or high risk of bias, respectively. Scores of eleven or ten, five to nine, and four or less were considered low, moderate, and high risk of bias in cohort studies, respectively. In a randomised controlled trial, the risk of bias was considered high for scores less than six, moderate for scores six to eleven, and low for scores twelve or thirteen.

### Data analysis

A random-effects meta-analysis was used to pool the prevalence of exclusive breastfeeding across the included studies to estimate the overall prevalence in Ghana. The pooled prevalence and corresponding 95% confidence intervals were presented in a forest plot. The proportion of total variability that was due to between study heterogeneity was estimated using Higgins and Thompson’s I^2^ statistic [29]. The presence of publication bias was assessed through Egger’s test and visual examination of funnel plot. We performed sub-group analysis by locality (rural-urban residence), administrative regions, and geographical zone (coastal-middle belt [Greater Accra, Central, Western, Volta, Ashanti, Brong-Ahafo and Eastern Regions] and savannah/northern belt [Northern, Upper East and Upper West Regions] [30]) to investigate potential sources of heterogeneity. Were it was not clear whether a study setting was a rural or urban locality, we referred to the Ghana statistical services report for the region for guidance. To determine the factors associated with exclusive breastfeeding, data from the studies were summarised in tables, and the summarised data were synthesised and integrated to produce summary statements. Stata version 17 was used for all statistical analysis.

## Results

### Characteristics of included studies

**Fig. 1:**
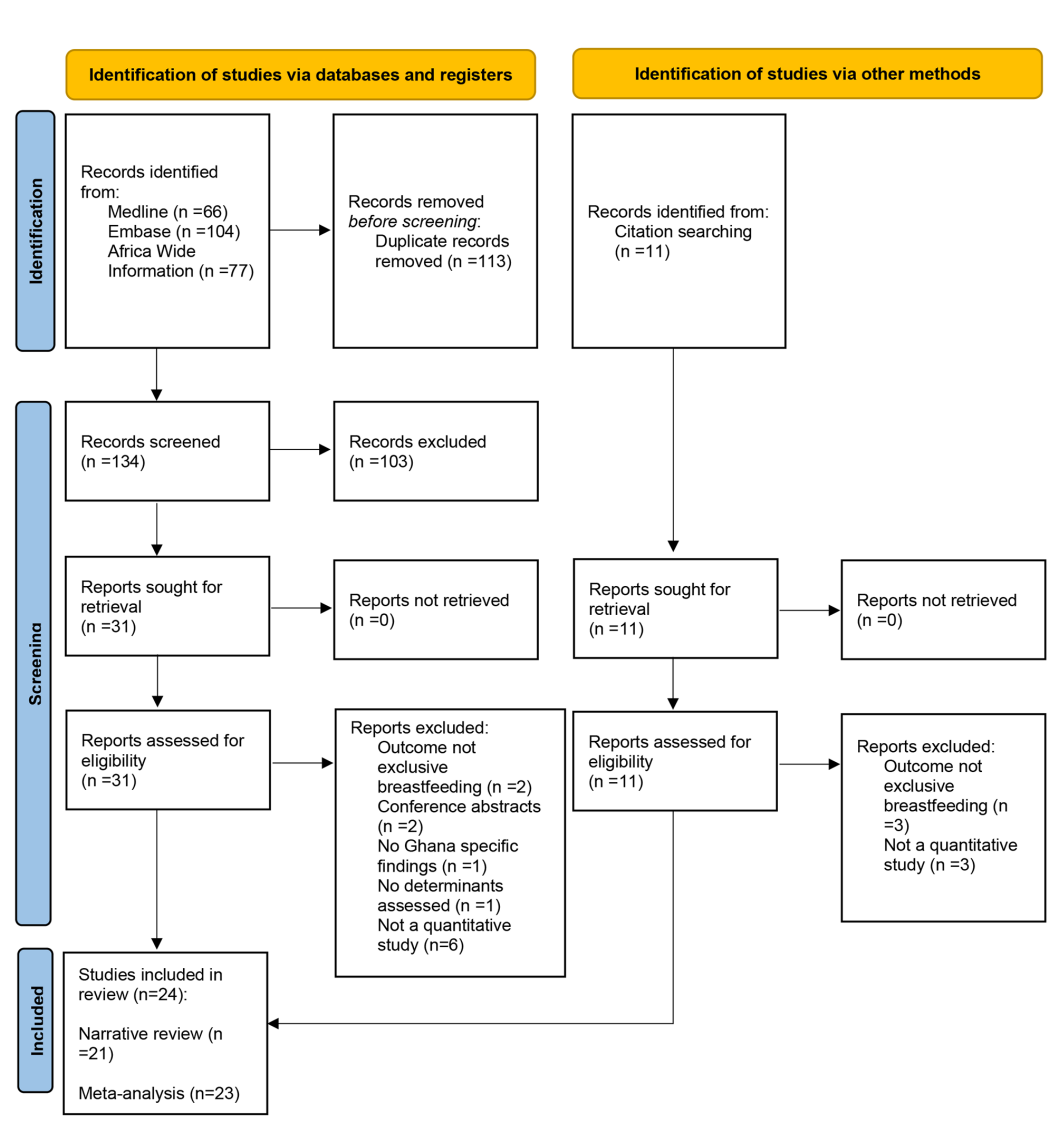
Flow diagram showing study selection

The PRISMA flow chart in Fig. 1 illustrates the study selection procedure. The database searches yielded 247 potential articles, and the hand search turned up 11 additional articles. In total, 42 articles were eligible for full-text screening after removing duplicates (n = 113) and screening titles and abstracts of 145 articles. Of the 42 articles that underwent full-text screening, 24 were included in the systematic review. Table 1 summarises the characteristics and summary findings of the included studies. Twenty-one of the included studies were cross-sectional, two were prospective cohort studies, and one was a randomised controlled trial. The sample sizes of the quantitative studies varied between 108 and 1870, with a total sample size of 8740 participants included in this review. Most study participants were recruited from healthcare facilities. The 24 included studies were published between 2005 and 2021, with 92% (n = 22) published in the last decade. Overall, the included studies were conducted in seven of the ten former regions of Ghana: Greater Accra region (n = 6), Northern region (n = 5), Ashanti region (n = 5), Central region (n = 2), Eastern region (n = 1), Upper West region (n = 1), and Volta region (n = 1). Three studies were nationally representative using data from the Ghana Demographic and Health Survey [12, 31, 32].

### Critical appraisal of included studies

We used descriptive statistics (counts and percentages) to summarise the scores of each study on the JBI tools. Of the 21 cross-sectional studies (Supplementary Table 3), ten (47.6%) had a low risk of bias, while 11 (52.4%) had a moderate risk of bias. None had a high risk of bias. All the cross-sectional studies appropriately measured the exposures and outcome, and nearly all employed an appropriate statistical method. The two cohort studies each scored 10 on the 11-item JBI tool indicating a low risk of bias (Supplementary Table 4). Nonetheless, the cohort studies lacked a detailed explanation of the methods used to address incomplete follow-up. The risk of bias in the randomised controlled trial was moderate, as measured by a score of 11 on the 13-item JBI tool (Supplementary Table 5). Outcome assessors were not blinded to the allocation of interventions in the randomised controlled trial.

### Prevalence of exclusive breastfeeding in Ghana

**Fig. 2:**
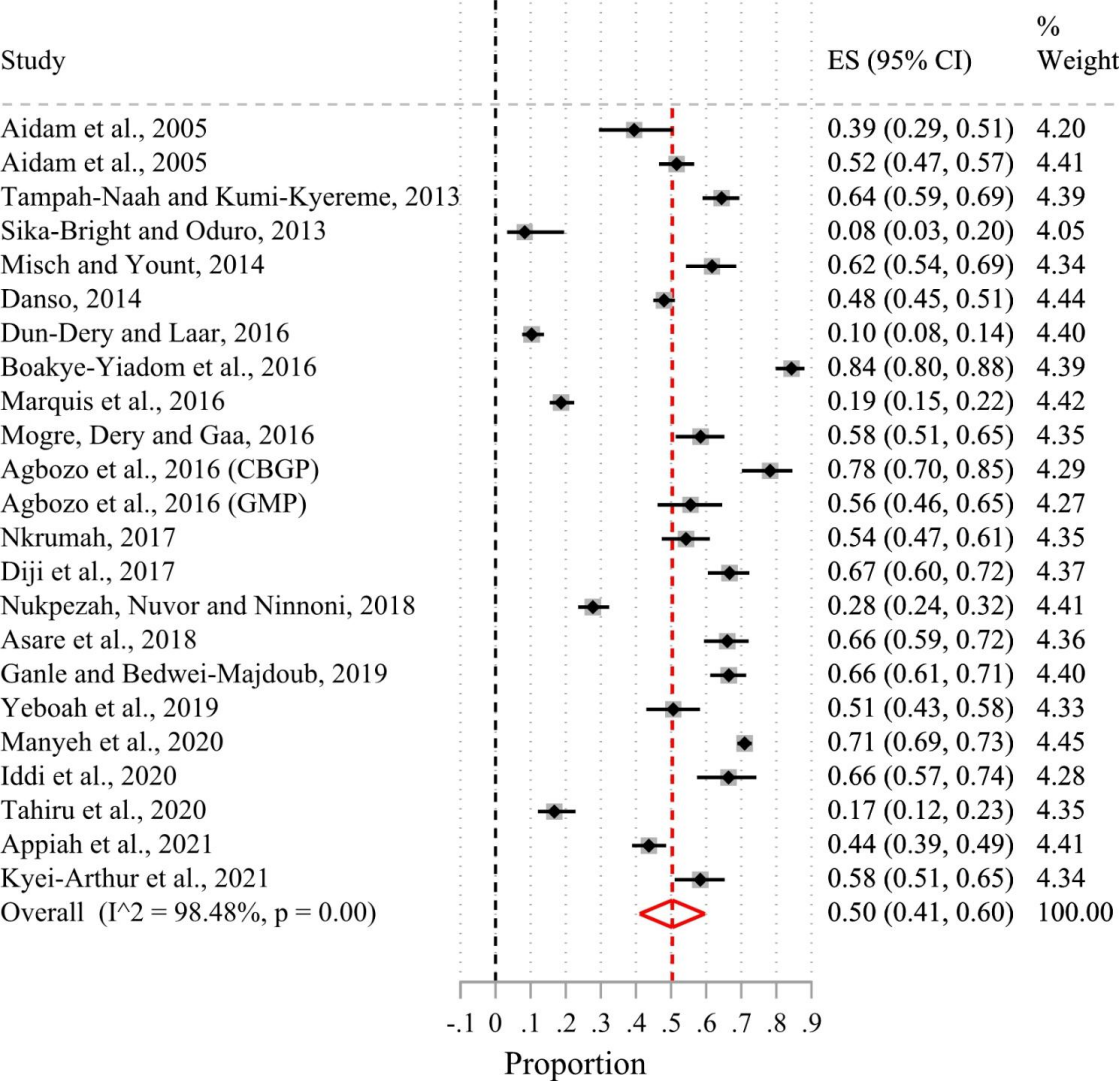
Forest plot of the prevalence of exclusive breastfeeding among children younger than six months in Ghana

**Fig. 3:**
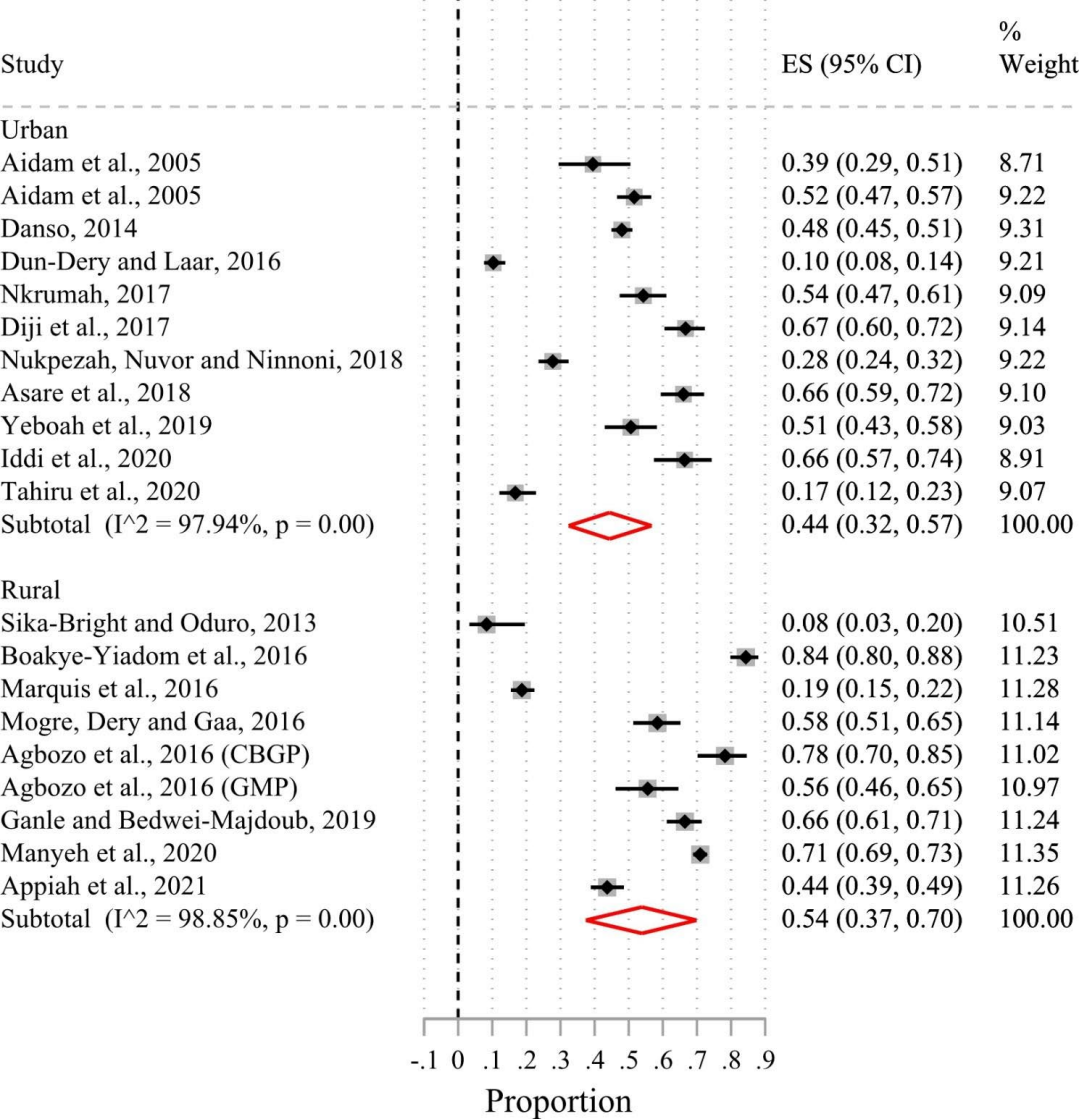
Forest plot of the prevalence of exclusive breastfeeding by rural and urban residence of Ghana

**Fig. 4:**
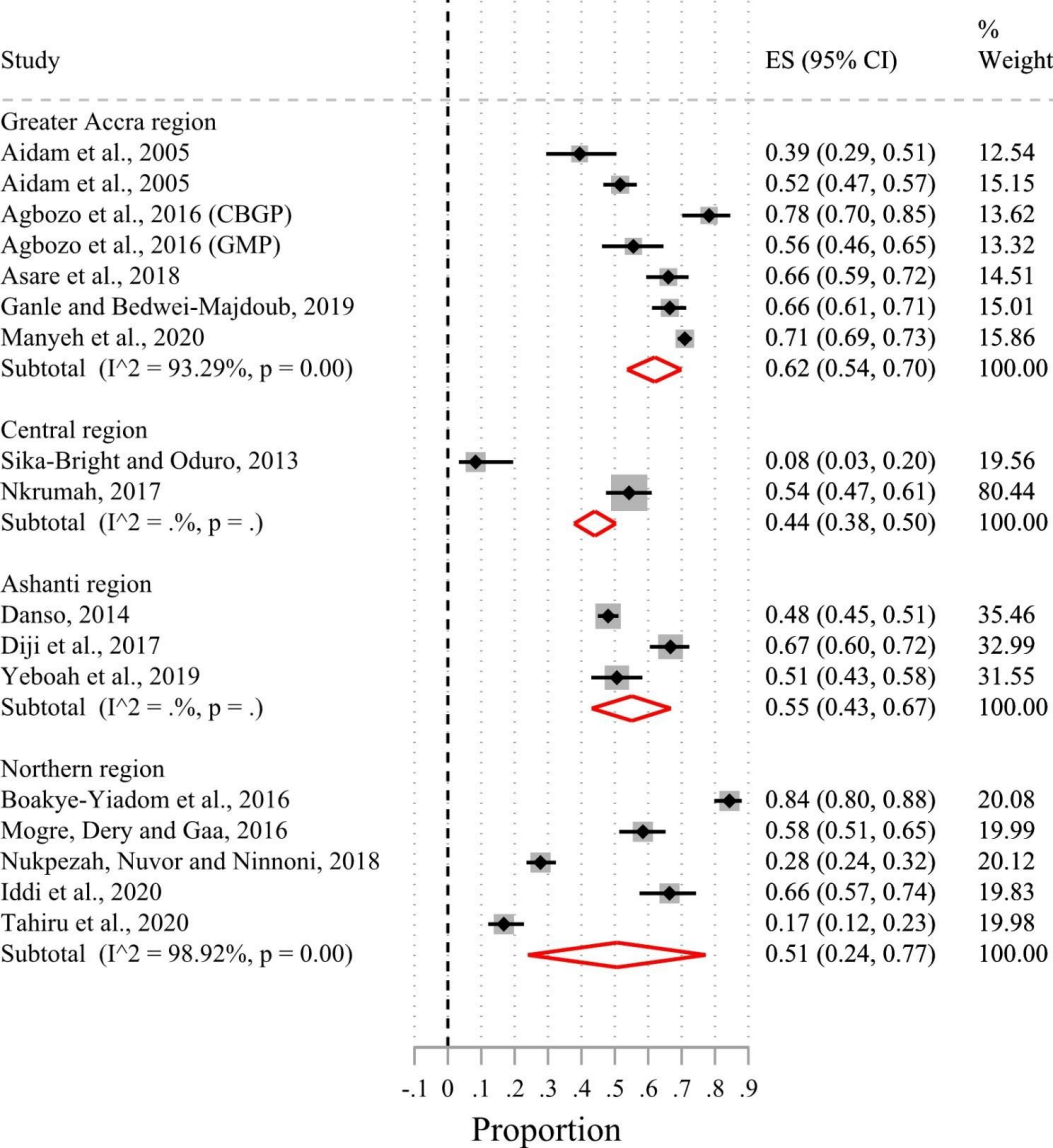
Forest plot of the prevalence of exclusive breastfeeding by administrative regions of Ghana

**Fig. 5:**
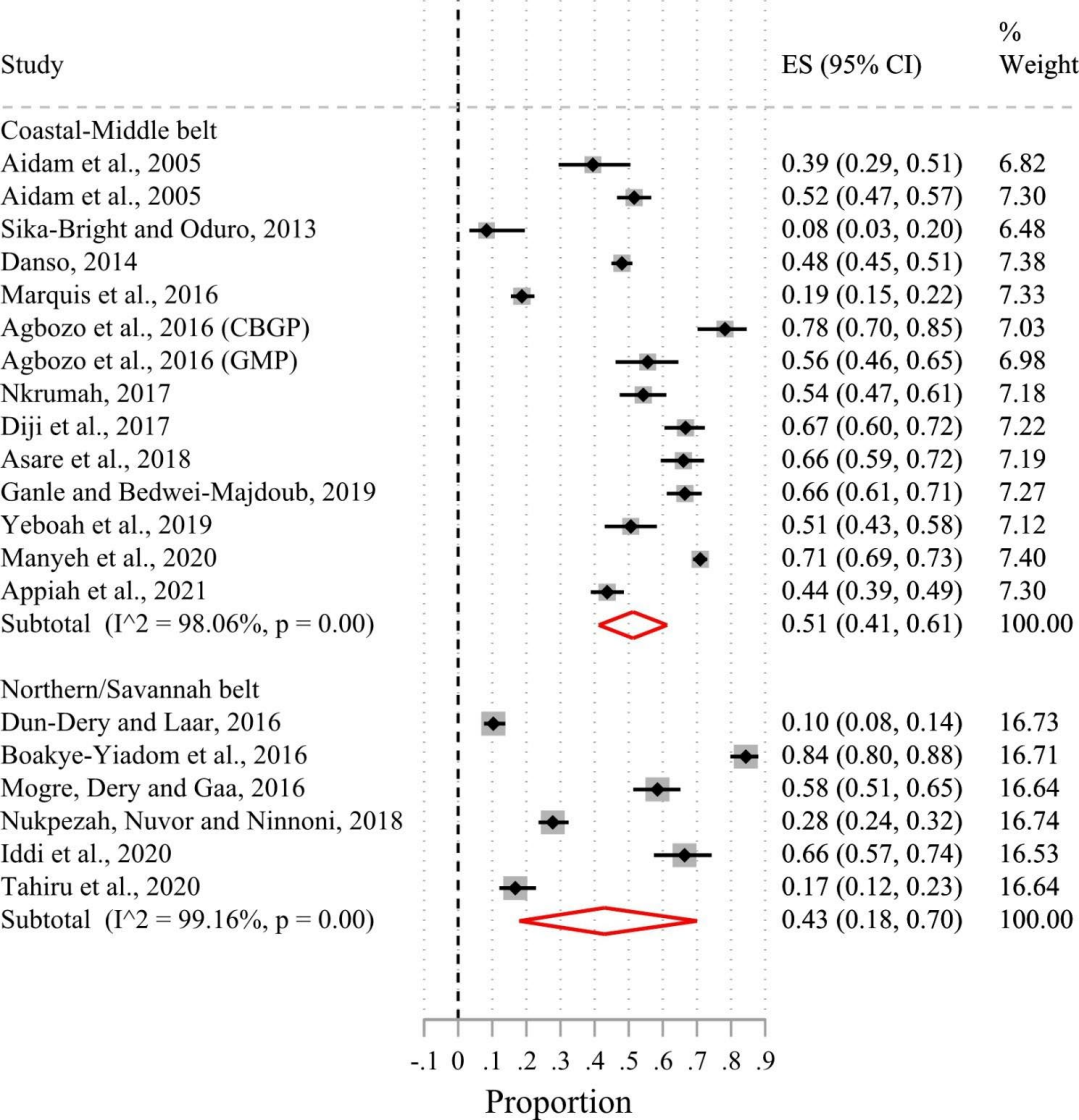
Forest plot of the prevalence of exclusive breastfeeding by geographical zones of Ghana

Of the 24 included studies, 23 had suitable data for a meta-analysis. Based on the random-effects meta-analysis of the 23 studies (Fig. 2), the pooled prevalence of exclusive breastfeeding in the first six months of life in Ghana was 50% (95%CI 41.0–60.0%; I^2^ = 98.5%). There was no evidence of publication bias after examining the funnel plot (Supplementary Fig. 1) and based on Egger’s test (p-value = 0.56). Sub-group analysis by urban-rural residence showed that the prevalence of exclusive breastfeeding in the first six months after birth was 44% (95%CI 32.0–57.0%; I^2^ = 97.9%) in urban areas and 54% (95%CI 37.0–70.0%; I^2^ = 98.9%) in rural areas (Fig. 3). Figure 4 shows that the pooled prevalence of exclusive breastfeeding at the level of the administrative regions was 62% (95%CI 54.0–70.0%; I^2^ = 93.3%) in the Greater Accra region, 55% (95%CI 43.0–67.0%; I^2^ = 0%) in the Ashanti region, 51% (95%CI 24.0–77.0%; I^2^ = 98.9%) in the Northern region, and 44% (95%CI 38.0–50.0%; I^2^ = 0%) in the Central region. Heterogeneity in prevalence estimates was lower among studies conducted in the Greater Accra region than those from the other regions. The pooled prevalence based on geographic zone sub-group analysis indicated that the prevalence of exclusive breastfeeding was higher (51%; 95%CI 41.0–61.0%; I^2^ = 98.1%) in the coastal-middle belt than in the northern/savannah belt (43%; 95%CI 18.0–70.0%; I^2^ = 99.2) (Fig. 5).

## Determinants of exclusive breastfeeding in Ghana

Of the 24 included studies, 21 had suitable data for narrative synthesis. The determinants of exclusive breastfeeding identified in the 21 studies were grouped into maternal and paternal factors and infant factors (Table 2).

### Maternal and paternal factors

The maternal and paternal factors associated with exclusive breastfeeding in the first six months were categorised into (1) sociodemographic factors, (2) workplace-related factors and influences, (3) obstetric and healthcare factors, and (4) feeding practices or decisions.

#### Sociodemographic factors

We found that exclusive breastfeeding in the first six months after birth was more likely among women aged 20 or older [33–35], women who lived in a large household or with many children [32, 33, 36], self-employed women [37], unemployed women [35] rural women with higher education [38], women from the Volta region [39], and women who owned a house [40].

However, exclusive breastfeeding in the first six months was less likely among unmarried women [41], women residing in urban areas [17], urban women with higher education [34], women living in fishing districts [33], women of the Akan or northern ethnic groups [34, 36], women who have experienced intimate partner violence [31], those without access to radio [42], women with a partner who had primary education [32], and women with a partner who desired more children [32].

#### Workplace related factors

In the first six months after giving birth, women who worked as artisans [33] and those given less than three months of maternity leave [43] were less likely to practice exclusive breastfeeding.

#### Obstetric and healthcare factors

Three studies reported that women who delivered in health facilities were more likely to practice exclusive breastfeeding in the first six months than those who delivered outside a health facility [39, 40, 44]. In two studies, an increase in antenatal care visits was positively associated with exclusive breastfeeding in the first six months after birth [44] [32]. Two studies found that women who had normal delivery were more likely to practice exclusive breastfeeding than those delivered through caesarean Section. [35, 43]. Also, mothers who had healthy nipples were more likely to exclusively breastfeed for a longer duration than those who had sore nipples [35]. On the other hand, women who made less than four antenatal care visits [17] and those who were HIV positive [36] were less likely to practice exclusive breastfeeding. In one study, women who had 3–4 previous deliveries were less likely to practice exclusive breastfeeding [35].

#### Feeding practices, decisions, and support

In two studies, women with higher knowledge of exclusive breastfeeding were more likely to practice exclusive breastfeeding than those with inadequate knowledge [38, 44]. Likewise, women who received counselling on breastfeeding were more likely to exclusively breastfeed their children in the first six months after birth [13, 45]. Also, women who participated in mother-to-mother support groups were more likely to practice exclusive breastfeeding [44]. One study reported that women who planned to exclusively breastfeed on delivery or had positive attitudes towards exclusive breastfeeding were more likely to exclusively breastfeed in the first six months after birth [40]. Not knowing when to start complementary feeding or believing it is appropriate to introduce complementary feeds at six months was positively associated with exclusive breastfeeding in the first six months [13].

On the other hand, counselling on complementary feeding [13], formula feeding recommendations from health workers [43], non-awareness of exclusive breastfeeding [13], short duration of breastfeeding [13, 41], perceived inability to produce enough breastmilk [42], lack of support from family [17], advise from support person to formula feed [43], and outside pressure to introduce other foods [17] were associated with a lower likelihood of exclusive breastfeeding in the first six months after birth.

### Infant factors

We found in two studies that a child’s average size at birth or lower weight-for-age-z score was associated with an increased likelihood of exclusive breastfeeding [36, 39]. Infants younger than three months [38] and those who never fed from a bottle [13] were more likely to breastfeed exclusively in the first six months after birth. In contrast, two studies found that older infants were less likely to be exclusively breastfed than younger infants [32, 37]. In addition, one study [42] reported that children admitted to Neonatal Intensive Care Units (NICU) were less likely to exclusively breastfeed in the first six months.

**Table 1:** Characteristics and summary of findings of included studies

Author(s), year of publication.	Study location and residency type	Study design and sampling technique	Description of study participants (n)	Measurement of EBF	Factors associated with EBF/Comments
Agbozo et al., 2016(50) **Duration**: Unclear	**Location**: Nsakina and Dom Sampaman, Greater Accra region. **Residence**: Rural	**Design**: Cross-sectional **Sampling**: Unclear	Participants were caregiver–child pairs enrolled in either a community-based growth promotion (CBGP) programme or a growth monitoring and promotion (GMP) programme. The two programmes were delivered by community health nurses to promote optimal infant and young child feeding (IYCF). Caregivers who received child welfare services in either the CBGP (N = 124) or GMP (N = 108) between January and March 2012 were invited to participate in a follow-up study.	The food frequency questionnaire used during the 2008 Ghana demographic and health survey was used to collect a 3-day information on habitual dietary intakes of the children. However, it is unclear the specific breastfeeding questions that were asked.	The study separately reported the prevalence of exclusive breastfeeding at six months for children in the CBGP and GMP. Determinants of exclusive breastfeeding were not assessed (see Fig. 1).
Aidam et al., 2005(40) **Duration of study**: May to August 2000	**Location**: Ablekuma, Greater Accra region. **Residence**: unclear	**Design**: cross-sectional study **Sampling**: convenience sampling method	Women with infants 0–6 months attending maternal and child health (MCH) clinics in Accra (n= 376)	Mothers were asked if eight categories of liquid, mushy or solid foods had been given to the child since birth. The age at which these foods were first introduced was also recorded. A 24-hour recall was then used to verify if any of these same categories of liquids and foods had been given to the child within the past 24 h.	**Facilitators**: planned EBF on delivery, delivery at a hospital/polyclinic, women living in their own houses, and positive attitude towards EBF.
Aidam et al., 2005(45) **Duration of study**: Unclear	**Location**: Tema municipality, Greater Accra region. **Residence**: Urban	**Design**: Randomised controlled trial **Sampling**: Unclear	Pregnant women in their last trimester of gestation, attending prenatal clinics at two hospitals in Tema Township, planned to stay in Tema or Ashiaman for at least six months after delivery, and their term infants delivered singleton at 36–44 weeks gestation with normal birth weight and Apgar scores ≥ 6 (n = 123).	Data were collected on the onset of lactation, current breastfeeding status, total number of breastfeeds over the past 24 h, recall of liquid or semisolid foods introduced to the infant over the previous month and the past 24 h, and frequency and age of introduction of specific liquids or foods and reasons for their introduction.	**Facilitators**: lactation counselling
Appiah et al., 2021(13) **Duration of study**: October 2018 – February 2019	**Location**: Hohoe, Ho West, and Ketu South, Volta Region. **Residence**: Unclear	**Design**: cross-sectional study **Sampling**: Multistage sampling	Mothers living in the selected districts with children aged 0 to 59 months (n = 396).	Mothers were asked when they started breastfeeding after birth, if other foods were given on the first day after birth, the number of times the child was breastfed in a day, and whether the child was ever fed from a bottle with a nipple. The questionnaire also contained questions on whether the mother has ever heard about EBF and the appropriate age of a child when the mother thinks she can start complementary feeding, and the months the child should breastfeed before being weaned off from the breast.	**Facilitators**: being a Muslim or Traditionalist, counselled on exclusive breastfeeding, children who were never fed from a bottle with a nipple, not knowing when to start complementary feeding or the belief that it is appropriate to start complementary feeding at 1–3 months or six months, **Barriers**: counselling on complementary feeding, non-awareness of exclusive breastfeeding, short duration of breastfeeding,
Asare et al., 2018(34) **Duration of study**: May to June 2016	**Location**: Tema East Sub-Metropolitan area, Greater Accra region **Residence**: Unclear	**Design**: cross-sectional study **Sampling**: Simple random sampling	Participants were mothers aged 15–49 years attending Child Welfare Clinic at Manhean Health Centre with infants and young children aged 0–24 months (n = 355)	Mothers were asked if they had breastfed the child in the past 24 h, how soon after birth the child was given breastmilk, and whether they were fed with the first breastmilk. Also, information was collected on all foods or liquids given to the child in the last 24 h before the interview and whether feeding was done using a feeding bottle.	**Facilitators**: Mothers aged 20–24, 25–29, and 30–34 **Barriers**: Mothers with tertiary education and those from ethnic groups in northern Ghana
Ayawine and Ae-Ngibise, 2015(41) **Duration of study**: June 2009 to September 2010	**Location**: Abuakwa and Barekese, Ashanti region **Residence**: Peri-urban and rural	**Design**: cross-sectional study **Sampling**: Systematic, random, and purposive sampling methods	Nursing mothers attending child welfare clinics in six communities located in the two sub-districts under study (n = 300).	Unclear	**Barriers**: unmarried mothers, breastfeeding for three months
Boakye-Yiadom et al., 2016(14) **Duration of study**: February 2014 to June 2014.	**Location**: West Mamprusi district, Northern region **Residence**: Unclear	**Design**: cross-sectional study **Sampling**: Multistage sampling	Participants were breastfeeding mothers in their seventh month after delivery with infants 0–6 months of age and residing in the West Mamprusi district (n = 300).	Unclear	**Facilitators**: Household wealth index, antenatal care attendance, knowledge of exclusive breastfeeding, place of delivery, and mother-to-mother support group.
Danso, 2014(48) **Duration of study**: Unclear	**Location**: Kumasi metropolis, Ashanti Region **Residence**: Unclear	**Design**: cross-sectional study **Sampling**: Purposive and random sampling	The study population consisted of professional working mothers aged 40 or younger who were in full-time employment and working in the Kumasi metropolis of Ghana (n = 1000).	Unclear	**Barriers**: Maternal employment, influence of family members*
Diji et al., 2017(37) **Duration of study**: January to March 2015	**Location**: Kumasi South, Ashanti Region **Residence**: Unclear	**Design**: cross-sectional study **Sampling**: Simple random sampling	Participants were mothers with healthy infants aged 3–9 months (n = 240)	Unclear	**Facilitator**: Mother self-employed **Barriers**: increasing age of the child
Dun-Dery and Laar, 2016(43) **Duration of study**: January to July 2015	**Location**: Wa, Upper West region **Residence**: Urban	**Design**: cross-sectional study **Sampling**: systematic random sampling	Professional working mothers resident in Wa (n = 369).	Exclusive breastfeeding rate was measured using three questions: (1) How many months did you breastfeed the child on only breastmilk, (2) At what age of the child did you introduce other foods and drinks, and (3) Did you give the child anything to eat or drink apart from breast milk before the child was six months old.	**Facilitators**: Normal delivery **Barriers**: Less than three months of maternity leave, formula feeding recommendation from health workers, and advice by a support person to formula feed.
Ganle and Bedwei-Majdoub, 2019(17) **Duration of study**: January 2017 to May 2018	**Location**: Shai-Osudoku District, Greater Accra region **Residence**: Rural	**Design**: Prospective cohort study **Sampling**: Convenient sampling method	Mothers aged 15–49 who had normal and full-term delivery at the Shai-Osudoku District Hospital (n = 322).	The exact timing of exclusive breastfeeding initiation and discontinuation was collected from mothers through monthly follow-up visits via telephone and home visits in some cases.	**Barriers**: four or fewer antenatal visits, lack of support from family, outside pressure to provide other food to the baby, and living in an urban area.
Iddi et al., 2020(51) **Duration of study**: Unclear	**Location**: Tamale, Northern Region **Residence**: Unclear	**Designed**: Cross-sectional study **Sampling**: systematic and convenient sampling.	Women who worked as nurses in Tamale and had babies younger than six months were asked to take part in the study (n = 125).	Mothers were asked to complete a questionnaire about their exclusive breastfeeding practices and engaged in focused group discussions to record their diverse experiences with the practice of exclusive breastfeeding in the workplace setting, as well as the facilitators and barriers of exclusive breastfeeding in the work environment.	The prevalence of exclusive breastfeeding was reported (see Fig. 1).
Kyei-Arthur et al., 2021(32) **Duration of study**: Unclear	**Location**: Nationally representative sample (2014 DHS) **Residence**: Rural and urban	**Design**: cross-sectional study **Sampling**: Multistage sampling	Infants less than six months (exclusively breastfed or not) who had both maternal and paternal characteristics (n = 180).	Exclusive breastfeeding was measured using several food items such as breastmilk, water, liquids, milk, and solid food. Infants fed only on breast milk 24 h before the survey were considered exclusively breastfed.	**Facilitators**: increasing number of children ever born, increase in antenatal care visits **Barriers**: Paternal primary education, paternal desire for more children, father being a Muslim, older age of infants,
Manyeh et al., 2020(33) **Duration of study**: January 1, 2011, and December 31, 2013,	**Location**: Shai-Osudoku and Ningo-Prampram districts, Greater Accra region **Residence**: Rural	**Design**: cross-sectional study **Sampling**: Unclear	All women residents in the Osudoku and Ningo-Prampram districts who were registered in the Dodowa HDSS and gave birth between January 1, 2011, and December 31, 2013 (n = 1870).	Breastfeeding practices from birth to the first six months of life of the index child were collected from mothers retrospectively at six months	**Facilitators**: Mothers aged 25–29 and 30 + years, household size of more than five members **Barriers**: mothers who are artisans, residence in a fishing district
Marquis et al., 2016(36) **Duration of study**: 2003 and 2008	**Location**: Yilo Krobo and Manya Krobo Districts, Eastern region **Residence**: unclear	**Design**: Prospective cohort study **Sampling**: Unclear	Participants were pregnant women (at the time of enrolment) who took part in a VCT pre-test counselling, delivered a live infant with no birth defects, and had no clinical or physical ailments that would limit their ability to care for their infant (n = 482).	Mothers were visited at home twice weekly and asked about their infant’s breastmilk intake, non-milk liquids, animal-based milk, infant formula and solid and semisolid foods during the previous days since the last visit.	**Facilitators**: Lower weight-for-age z-score and higher number of children under five years in a household. **Barriers**: Mother’s HIV-positive status and Akan or northern ethnic groups.
Mensah et al., 2017(46) **Duration of study**: Unclear	**Location**: Sekere-South District, Ashanti region **Residence**: peri-urban	**Design**: cross-sectional study **Sampling**: Convenient sampling method	Nursing mothers attending postnatal care at the postnatal clinics in all the 13 health facilities with child welfare clinics (both public and private) in the Sekere-South District (n = 380)	Not available	Mothers level of education, religion, ethnicity, type of employment, number of births, sources of information about exclusive breastfeeding, steps taken by mothers who said they did not have enough breastmilk, and medical conditions.*
Misch and Yount, 2014(31) **Duration of study**: Unclear	**Location**: Nationally representative sample (DHS survey) **Residence**: Rural and urban	**Design**: cross-sectional study **Sampling**: Multistage sampling	Women 15–49 years who were matched to an infant less than six months old, who had completed an IPV module with questions on psychological, physical, and sexual IPV, who also had answered questions about breastfeeding their youngest child, and who had completed data for all outcomes and exposure variables (n = 173).	To determine exclusive breastfeeding, mothers were asked about liquids or foods their infant had consumed in the prior 24 h.	**Barriers**: Sexual IPV victimisation
Mogre, Dery and Gaa, 2016(38) **Duration of study**: January to July 2015	**Location**: Tuna, Northern Region **Residence**: Rural	**Design**: cross-sectional study **Sampling**: Nonprobability sampling	Mothers and/or caregivers that attended the antenatal clinic of the Tuna Health Centre with apparently healthy infants aged 0–6 months during the study period (n = 190)	Mothers’ practice of EBF was assessed with a recall of EBF in the last 24 h, mode of breastfeeding, who gave and what kind of food was given to the baby in the mother’s absence, the introduction of liquids	**Facilitators**: Higher maternal education, infants younger than three months, and higher maternal knowledge of EBF.
Nkrumah, 2017(49) Duration: July to November 2015	**Location**: Effutu Municipality, Central Region **Residence**: Urban	**Design**: Cross-sectional study **Sampling**: Universal sampling technique	Participants were mother-infant pairs attending a community-based Child Welfare Clinic organized by the Effutu Municipal Reproductive and Child Health Unit between July and November 2015. Mothers with infants up to seven months of age were interviewed (n = 225).	A 24 h self-recall method was used to assess exclusive breastfeeding and mothers’ breastfeeding frequency.	Sector of work*
Nukpezah, Nuvor and Ninnoni, 2018(47) **Duration of study**: unclear	**Location**: Tamale, Northern Region **Residence**: Urban	**Design**: cross-sectional study **Sampling**: Purposive, quota, and simple random sampling	Lactating mothers of the Tamale Metropolitan area with a child of at least six months and at most two years at the time of the research and attending a child welfare clinic (CWC) in Tamale Teaching Hospital, Tamale West Hospital or Tamale Central Hospital (n = 393).	Unclear	Sex of child, and knowledge on whether EBF allow child spacing and reduces the risk of breast cancer*
Sika-Bright and Oduro, 2013(18) **Duration**: September and December 2012	**Location**: Duakor, Central region **Residence**: Rural	**Design**: explorative research design **Sampling**: Snowball and purposive sampling	Mothers with children aged six months to two years (n = 48).	Breastfeeding mothers were asked about their breastfeeding practices during the first six months of their baby’s life and the factors and attitudes of significant others that informed their breastfeeding decisions and practices.	This qualitative study reported the prevalence of exclusive breastfeeding among children under six months (see Fig. 1).
Tahiru et al., 2020(42) **Duration of study**: Unclear	**Location**: Tamale Metropolis, Northern Region. **Residence**: Urban and rural	**Design**: cross-sectional study **Sampling**: consecutive and purposive sampling	Participants were mothers with twins aged 6–23 months in the Tamale Metropolis (n = 185).	Mothers were asked whether the children were given any feed aside from breastmilk in the first six months of life and the age (in months) at which water or other liquids was given.	**Barriers**: Perceived inability to produce enough breast milk, no access to radio, child admission into neonatal intensive care unit (NICU).
Tampah-Naah and Kumi-Kyereme, 2013(39) **Duration of study**: Unclear	**Location**: Nationally representative sample (2008 DHS) **Residence**: Rural and urban	**Design**: cross-sectional study **Sampling**: Multistage sampling	Women aged 15–49 in selected households in the ten regions of Ghana (n = 316).	Maternal recall of breastfeeding practices in the 24 h preceding the survey interview.	**Facilitators**: delivery in a government health facility, average size of child at birth, mother from Volta region
Yeboah et al., 2019(35) **Duration**: January and February 2017	**Location**: Kumasi, Ashanti region **Residence**: Unclear	**Designed**: Cross-sectional study **Sampling**: Simple random sampling	Participants were women with infants aged 6–24 months attending maternal and child health services in five government health care facilities (hospitals and health centres) in the Kumasi Metropolis (n = 160)	Women were asked if they practised exclusive breastfeeding in the first six months of life and the sources of information on exclusive breastfeeding practice.	**Facilitators**: Maternal age 30–49 years, with normal delivery, maternal unemployment, and absence of sore nipple **Barriers**: previous 3–4 deliveries

*Study did not adjust for potential confounders, and the direction of association was unclear

**Table 2:** Determinants of exclusive breastfeeding in the first six months after birth in Ghana

		Factors associated with increased likelihood of exclusive breastfeeding in the first six months after birth	Factors associated with a lower likelihood of exclusive breastfeeding in the first six months after birth
Themes	Sub-themes	Codes	Codes
1. Maternal factors	Sociodemographic factors	1. Mothers aged 25–29 and 30 + years/ Mothers aged 20–24(33)(34)(35) 2. Mother from the Volta region(39) 3. Household size of more than five members/ higher number of children under five years/ increasing number of children ever born(33)(36)(32) 4. Women living in their own houses(40) 5. Rural women with higher education(38) 6. Household wealth index(44) 7. Mother self-employed(37) 8.Maternal unemployment(35)	1. Unmarried mothers(41) 2. Living in an urban area(17) 3. Residence in a fishing district(33) 4. Akan or northern ethnic groups/ ethnic groups in northern Ghana(36)(34) 5. Urban mothers with tertiary education(34) 6. Paternal primary education(32) 7. Sexual IPV victimisation(31) 8. No access to radio(42) 9. Paternal desire for more children(32)
	Workplace related factors		1. Mothers who were artisans (33) 2. Less than three months of maternity leave(43)
	Obstetric and Healthcare factors	1. Delivery in a health facility(39)(40)(44) 2. Normal delivery(43)(35) 3. Antenatal care attendance/ increase in antenatal care visits (44)(32)	1. Four or fewer antenatal visits(17) 2. Mother’s HIV-positive status(36) 3. Three to more previous deliveries(35)
	Feeding practices, decisions, and support	1. Planned EBF on delivery/positive attitude towards EBF(40) 2. Higher maternal knowledge of EBF(38)(44) 3. Counselled on exclusive breastfeeding/ lactation counselling (13)(45) 4. Not knowing when to start complementary feeding or the belief that it is appropriate to start complementary feeding at 1–3 months or six months(13) 5. Mother-to-mother support group(44) 6. Absence of sore nipple(35)	1. Breastfeeding for three months/Short duration of breastfeeding (41) (13) 2. Counselling on complementary feeding(13) 3. Non-awareness of exclusive breastfeeding(13) 4. Perceived inability to produce enough breast milk(42) 5. Formula feeding recommendation from health workers(43) 6. Lack of support from family(17) 7. Advise by a support person to formula feed(43) 8. Outside pressure to provide other food to the baby(17)
2. Infant factors	Infant characteristics	1. Average size of child at birth/lower weight-for-age z-score (39)(36) 2. infants younger than three months(38) 3. Children who were never fed from a bottle with a nipple(13)	1. Increased age of the child/ older age of infants(37)(32) 2. Admission of a child into a neonatal intensive care unit (NICU)(42)

## Discussion

We reviewed the current evidence on the prevalence and determinants of exclusive breastfeeding in the first six months of life. To our knowledge, this is the first systematic review and meta-analysis on this subject in Ghana. Overall, the prevalence of exclusive breastfeeding in the first six months of life in Ghana was 50%. Subgroup analysis revealed that the prevalence is higher in rural areas than in urban areas. Our estimated prevalence of exclusive breastfeeding in Ghana is comparable to the reported prevalence in most West and Central African countries [52]. However, it is lower compared to prevalence in most East and Southern African countries [52]. These variations in exclusive breastfeeding rates are likely a result of regional and cultural variations in breastfeeding policies, practices, and expectations across sub-Saharan Africa. Furthermore, although our estimated prevalence for Ghana is lower than the World Health Assembly’s target of 70% prevalence of exclusive breastfeeding by 2030 [9], it is higher than the current global prevalence (44%) of exclusive breastfeeding [53] and the overall prevalence of exclusive breastfeeding (41%) for sub-Saharan Africa [52].

In contrast to our findings, a systematic review of 25 studies from nineteen developing countries found that older women were less likely to practise exclusive breastfeeding [54]. Unlike the current review, the earlier review included studies from diverse cultures and compared older women to younger non-adolescent women, which may explain the contradictory findings. Nevertheless, in line with our findings, a Brazilian systematic review of 27 studies found that teenage mothers were less likely to practise exclusive breastfeeding [55]. Younger mothers may have a lower exclusive breastfeeding rate due to a possible lack of awareness of the benefits of exclusive breastfeeding, inadequate breastfeeding skills, and unpleasant and painful breastfeeding experiences [56–59]. Young mothers often introduce complementary foods earlier than recommended to avoid the unpleasant experiences and perceived physical changes associated with exclusive breastfeeding [60, 61].

Consistent with our findings, previous studies have identified self-employment, higher maternal education, and house ownership as facilitators for exclusive breastfeeding [54, 57, 62, 63]. These factors empower women, increase their self-efficacy, and promote health-seeking behaviour, including the ability to address the challenges of exclusive breastfeeding [38, 64]. Healthcare professionals must become familiar with these enabling factors to identify at-risk mothers during prenatal and postpartum care and provide prompt intervention and counselling. Additionally, several previous systematic reviews have reported on the positive influence of antenatal attendance, facility delivery, and normal vaginal delivery on exclusive breastfeeding in line with our findings [54, 55, 57, 65]. In a mixed-methods systematic review, Patil et al., found that caesarean delivery was associated with a 1.6 times higher risk of exclusive breastfeeding cessation than normal vaginal delivery [57]. Antenatal attendance improves pregnant women’s knowledge of the nutritional value of breastmilk as well as their attitude toward exclusive breastfeeding. This explains why this study found a higher likelihood of exclusive breastfeeding among women who attended more antenatal visits and had good knowledge of exclusive breastfeeding.

Unsurprisingly, several previous studies have reported positive associations between exclusive breastfeeding and adequate maternal knowledge of the benefits of exclusive breastfeeding, positive attitude toward breastfeeding [54], social support from friends and family [52, 56, 57], and peer counselling on exclusive breastfeeding [54, 57], which is consistent with our results. For example, in 2018, a meta-analysis of 27 randomised controlled trials found that mothers with breastfeeding support were more likely to practice exclusive breastfeeding than those without support [63]. The positive impact of family support may explain why women in larger households were more likely to practise exclusive breastfeeding in this study. In Ghanaian society, a large household generally includes other family members who may support and encourage breastfeeding.

Births out-of-wedlock are generally regarded as morally inappropriate in Ghanaian society, and mothers who are not married receive little assistance from their families and community [66, 67]. It is possible that insufficient family and community support contributed to the lower likelihood of exclusive breastfeeding among unmarried women in this study. Indeed, a lack of family support was identified as a major barrier to the practice of exclusive breastfeeding in the current review. Furthermore, we found that intimate partner violence was negatively associated with exclusive breastfeeding, which is consistent with the findings of Normann et al. [68]. Women exposed to intimate partner violence in any form and at any stage are at significant risk of terminating exclusive breastfeeding before the recommended duration of six months [68].

Exclusive breastfeeding practices are impacted by mothers return to work [26, 38]. When returning to work, mothers frequently leave their infants with family members, spend less time with them, and introduce complementary foods earlier than is ideal [71]. Therefore, it is not surprising that in the present review, exclusive breastfeeding was less likely among women who returned to work within three months of giving birth. Several earlier studies have reported similar results [54, 72, 73]. There is a need for institutions to improve their support of breastfeeding in the workplace. For instance, policies extending paid maternity leave might increase exclusive breastfeeding duration among working mothers. In line with our findings, a prior systematic review found that women in urban areas were more likely to terminate exclusive breastfeeding before six months than those in rural areas [57]. This can be attributed to urban dwellers early return to work, busy work schedules, and infant illness from expressed breastmilk [74].

Maternal HIV-positive status as a barrier to exclusive breastfeeding, as found in this review, can be attributed to poor policy dissemination, inadequate counselling, and mothers’ fear of passing the infection to their children through breastmilk [75]. Even though there is some risk of transmitting HIV through breastmilk [76], there is evidence suggesting a reduced viral load in women who adhere to antiretroviral drugs [77]. For instance, a Cochrane review reported a 29% reduction in HIV transmission through breastmilk within 18 months of breastfeeding [78].

When health professionals recommend infant feeding, mother’s and child’s best interest are considered. Therefore, it is surprising and unclear why health professionals in one of the studies under review [43] suggested complementary and formula feeding for infants younger than six months. It will be useful for future studies to investigate this practice. In agreement with our findings, a previous study found that women’s perceptions of insufficient breastmilk are linked to a lower likelihood of exclusive breastfeeding [54].

Our findings have implications for public health and clinical practice. A holistic approach is required to increase the prevalence of exclusive breastfeeding in the first six months of life and interventions should be multi-dimensional. For example, some inequalities derive the low prevalence of exclusive breastfeeding as demonstrated in this review and this may be addressed through the promotion of exclusive breastfeeding in all socioeconomic strata, localities, traditions, and languages. Guidelines should be culturally sensitive to provide a multicultural approach to breastfeeding counselling and education during antenatal and postnatal care and to ensure adherence. Accurate prenatal, perinatal, and postnatal educational interventions on exclusive breastfeeding, both within and outside health facilities, could correct misinformation on when to introduce complementary foods and discourage formula use in the first six months after birth. Family members and healthcare professionals can help adolescent mothers breastfeed exclusively for longer by providing breastfeeding support, encouragement, and guidance. Healthcare professionals must also provide adolescent mothers with adequate health education on the relevance and practice of exclusive breastfeeding. HIV-positive mothers need tailored counselling, education and support to promote exclusive breastfeeding. Public health authorities need to engage relevant stakeholders, including government and policymakers, to review the labour law to allow working mothers paid maternity leave for a minimum of six months. Policies should also be directed towards minimising the advertisements of breastmilk substitutes. Future reviews should focus on qualitative studies as evidence from these studies will likely uncover new understanding or perspectives on the factors that influence exclusive breastfeeding in Ghana.

Our study has several strengths. One major strength is that our findings are nationally representative as it includes studies from rural and urban areas of Ghana and across almost all the country’s administrative regions. In addition, the criteria for the search were not limited by region, language, or publication date. Despite the strengths, some limitations should be recognised when interpreting the findings of this study. One major limitation is that most of the studies were cross-sectional with the possibility of recall bias and measurement error due to the retrospective collection of breastfeeding data. In addition, some studies did not adequately control for known confounding variables. Another limitation of this study is that compared with an annual crude birth rate of 2.8, we report findings from 0.8% of babies born in Ghana annually. Even though most of the included studies adopted the WHO criteria for exclusive breastfeeding, the assessment and categorisation of exclusive breastfeeding varied across included studies. Three studies that analysed national surveys were not included in the subgroup analysis because it was not specific to a locality or administrative region. The regional subgroup analysis included only regions with at least two included studies.

## Conclusions

The prevalence of exclusive breastfeeding in Ghana is lower than the global target, though it is higher than the rates reported in most sub-Saharan African countries. We found that the determinants of exclusive breastfeeding in the first six months after birth included a range of sociodemographic, workplace-related, obstetric, and healthcare-related factors and feeding decisions and breastfeeding support available to mothers. Several infant characteristics were also identified as determinants of exclusive breastfeeding in the first six months after birth.

## Electronic supplementary material

Below is the link to the electronic supplementary material.

Supplementary Material 1

Supplementary Material 2

Supplementary Material 3

Supplementary Material 4

Supplementary Material 5

Supplementary Material 6

## Data Availability

All data generated or analysed during this study are included in this published article and its supplementary information files.
