# Supplementary material for: Systematic review and meta-analysis of the prevalence and determinants of exclusive breastfeeding in the first six months of life in Ghana
Source: BMC Public Health. 2023 May 19;23:920. doi: 10.1186/s12889-023-15758-w (PMC10199593; doi:10.1186/s12889-023-15758-w)
Supplement: Supplementary file 1 — Supplementary Material 1 [file 12889_2023_15758_MOESM1_ESM.docx]

**Supplementary Fig. 1 Funnel plot for publication bias**
