# Supplementary material for: Systematic review and meta-analysis of the prevalence and determinants of exclusive breastfeeding in the first six months of life in Ghana
Source: BMC Public Health. 2023 May 19;23:920. doi: 10.1186/s12889-023-15758-w (PMC10199593; doi:10.1186/s12889-023-15758-w)
Supplement: Supplementary file 2 — Supplementary Material 2 [file 12889_2023_15758_MOESM2_ESM.docx]

**Supplementary Table 1 Medline search plan**

|  | **Searches** |
| --- | --- |
| 1 | exclusive*.ti,ab. |
| 2 | exp Breast Feeding/ |
| 3 | 1 and 2 |
| 4 | (Breast* adj3 (alone or only or exclusive*)).ti,ab. |
| 5 | 3 or 4 |
| 6 | exp Ghana/ |
| 7 | Ghana*.ti,ab. |
| 8 | (Oti Region or Bono East Region or Ahafo Region or Bono Region or Brong-Ahafo Region or North East Region or Savannah Region or Western North Region or Western Region or Volta Region or Greater Accra Region or Eastern Region or Ashanti Region or Central Region or Northern Region or Upper East Region or Upper West Region).ti,ab. |
| 9 | 6 or 7 or 8 |
| 10 | 5 and 9 |
