# Supplementary material for: Systematic review and meta-analysis of the prevalence and determinants of exclusive breastfeeding in the first six months of life in Ghana
Source: BMC Public Health. 2023 May 19;23:920. doi: 10.1186/s12889-023-15758-w (PMC10199593; doi:10.1186/s12889-023-15758-w)
Supplement: Supplementary file 3 — Supplementary Material 3 [file 12889_2023_15758_MOESM3_ESM.docx]

**Supplementary Table 2 PECOS criteria for selection of studies**

| **Parameter** | **Inclusion criteria** | **Exclusion criteria** |
| --- | --- | --- |
| **Population** | Children aged 0-6 months | Non-Ghanaian children and children older than six months |
| **Exposure** | No restriction | No restriction |
| **Comparison** | No restriction | No restriction |
| **Outcome** | Exclusive breastfeeding | Exclusive breastfeeding after six months and other patterns of breastfeeding |
| **Study** | Cross-sectional, case-control, and cohort studies and randomised controlled trials | Systematic review, meta-analysis, protocols, dissertations |
