# Supplementary material for: Systematic review and meta-analysis of the prevalence and determinants of exclusive breastfeeding in the first six months of life in Ghana
Source: BMC Public Health. 2023 May 19;23:920. doi: 10.1186/s12889-023-15758-w (PMC10199593; doi:10.1186/s12889-023-15758-w)
Supplement: Supplementary file 4 — Supplementary Material 4 [file 12889_2023_15758_MOESM4_ESM.docx]

**Supplementary Table 3. Results of the critical appraisal of cross-sectional studies**

| **No** | **Author** | **Were the criteria for inclusion in the sample clearly defined?** | **Was the study setting described in detail?** | **Was the exposure measured in a valid and reliable way?** | **Were objective, standard criteria used for the measurement of the condition?** | **Were confounding factors identified?** | **Were strategies to deal with confounding factors stated?** | **Were the outcomes measured in a valid and reliable way?** | **Was appropriate statistical analysis used?** | **Score** |
| --- | --- | --- | --- | --- | --- | --- | --- | --- | --- | --- |
| 1 | Ayawine and Ae-Ngibise, 2015(1) | No | Yes | Yes | Yes | Unclear | Yes | Yes | Yes | 6 |
| 2 | Tampah-Naah and Kumi-Kyereme, 2013(2) | No | Yes | Yes | Yes. | Unclear | Yes | Yes | Yes | 6 |
| 3 | Manyeh et al., 2020(4) | Yes | Yes | Yes | Yes | Unclear | Yes | Yes | Yes | 7 |
| 4 | Dun-Dery and Laar, 2016(5) | Yes | Yes | Yes | Yes | Unclear | Yes | Yes | Yes | 7 |
| 5 | Aidam et al., 2005(6) | Yes | No | Yes | Yes | Unclear | No | Yes | Yes | 5 |
| 6 | Mensah et al., 2017(7) | Yes | No | Yes | Yes | No | No | Yes | Yes | 5 |
| 7 | Misch and Yount, 2014(9) | Yes | No | Yes | Yes | Unclear | Yes | Yes | Yes | 6 |
| 8 | Nukpezah, Nuvor and Ninnoni, 2018(10) | Yes | Yes | Yes | Yes | Unclear | No | Yes | Yes | 6 |
| 9 | Mogre, Dery and Gaa, 2016(11) | Yes | Yes | Yes | Yes | Unclear | Yes | Yes | Yes | 7 |
| 10 | Appiah et al., 2021(12) | Yes | Yes | Yes | Yes | Unclear | Yes | Yes | Yes | 7 |
| 11 | Asare et al., 2018(13) | Yes | Yes | Yes | Yes | Unclear | Yes | Yes | Yes | 7 |
| 12 | Diji et al., 2017(14) | Yes | Yes | Yes | Yes | Unclear | Yes | Yes | Yes | 7 |
| 13 | Danso, 2014 (49) | Yes | Yes | Yes | Yes | Unclear | No | Yes | Yes | 6 |
| 14 | Tahiru et al., 2020 (43) | Yes | Yes | Yes | Yes | Unclear | Yes | Yes | Yes | 7 |
| 15 | Boakye-Yiadom et al., 2016 (15) | Yes | Yes | Yes | Yes | Unclear | Yes | Yes | Yes | 7 |
| 16 | Kyei-Arthur et al., 2021 (33) | Yes | No | Yes | Yes | Yes | Yes | Yes | Yes | 7 |
| 17 | Nkrumah, 2017 (50) | Yes | Yes | Yes | Yes | Unclear | Yes | Yes | Yes | 7 |
| 18 | Yeboah et al., 2019 (36) | Yes | No | Yes | Yes | Unclear | Yes | Yes | Yes | 6 |
| 19 | Agbozo et al., 2014 (51) | No | No | Yes | Yes | Unclear | Yes | Yes | Yes | 5 |
| 20 | Failatu et al., 2020 (52) | Yes | Yes | Yes | Yes | No | No | Yes | No | 5 |
| 21 | Sika-Bright and Oduro, 2013 (19) | Yes | Yes | Yes | Not stated | No | No | Yes | No | 4 |
