# Supplementary material for: Systematic review and meta-analysis of the prevalence and determinants of exclusive breastfeeding in the first six months of life in Ghana
Source: BMC Public Health. 2023 May 19;23:920. doi: 10.1186/s12889-023-15758-w (PMC10199593; doi:10.1186/s12889-023-15758-w)
Supplement: Supplementary file 5 — Supplementary Material 5 [file 12889_2023_15758_MOESM5_ESM.docx]

**Supplementary Table 4 Results of the critical appraisal of cohort studies**

|  | **Were the two groups similar and recruited from the same population?** | **Were the exposures measured similarly to assign people to both exposed and unexposed groups?** | **Was the exposure measured in a valid and reliable way?** | **Were confounding factors identified?** | **Were strategies to deal with confounding factors stated?** | **Were the groups/participants free of the outcome at the start of the study (or at the moment of exposure)?** | **Were the outcomes measured in a valid and reliable way?** | **Was the follow up time reported and sufficient to be long enough for outcomes to occur?** | **Was follow up complete, and if not, were the reasons to loss to follow up described and explored?** | **Were strategies to address incomplete follow up utilized?** | **Was appropriate statistical analysis used?** | **Score** |
| --- | --- | --- | --- | --- | --- | --- | --- | --- | --- | --- | --- | --- |
| Ganle and Bedwei-Majdoub, 2019(3) | Yes | Yes | Yes | Yes | Yes | Yes | Yes | Yes | Yes | N/A | Yes | 10 |
| Marquis et al., 2016(8) | Yes | yes | Yes | Yes | Yes | Yes | Yes | Yes | Yes | Unclear | Yes | 10 |
