# Supplementary material for: Systematic review and meta-analysis of the prevalence and determinants of exclusive breastfeeding in the first six months of life in Ghana
Source: BMC Public Health. 2023 May 19;23:920. doi: 10.1186/s12889-023-15758-w (PMC10199593; doi:10.1186/s12889-023-15758-w)
Supplement: Supplementary file 6 — Supplementary Material 6 [file 12889_2023_15758_MOESM6_ESM.docx]

**Supplementary Table 5 Results of the critical appraisal of a randomised controlled trial**

|  | **Was true randomization used for assignment of participants to treatment groups?** | **Was allocation to treatment groups concealed?** | **Were treatment groups similar at the baseline?** | **Were participants blind to treatment assignment?** | **Were those delivering the treatment blind to treatment assignment?** | **Were treatment groups treated identically other than the intervention of interest?** | **Were outcome assessors blind to treatment assignment?** | **Were outcomes measured in the same way for treatment groups?** | **Were outcomes measured in a reliable way** | **Was follow up complete and if not, were differences between groups in terms of their follow up adequately described and analysed?** | **Were participants analysed in the groups to which they were randomized?** | **Was appropriate statistical analysis used?** | **Was the trial design appropriate and any deviations from the standard RCT design (individual randomization, parallel groups) accounted for in the conduct and analysis of the trial?** | **Score** |
| --- | --- | --- | --- | --- | --- | --- | --- | --- | --- | --- | --- | --- | --- | --- |
| Aidam et al., 2005 (46) | Yes | Yes | Yes | Yes | No | Yes | No | Yes | Yes | Yes | Yes | Yes | Yes | 11 |
